# Specific Detection of RHDV GI.1 and GI.2 by RT-LAMP-CRISPR/Cas12a Platform

**DOI:** 10.1155/tbed/3881457

**Published:** 2024-11-19

**Authors:** Mengting Wu, Mengmeng Chen, Rulong Qiu, Lei Ge, Zhiyu Fan, Bo Hu, Houjun Wei, Yiming Li, Fang Wang, Yanhua Song

**Affiliations:** ^1^Key Laboratory of Veterinary Biological Engineering and Technology Ministry of Agriculture, Institute of Veterinary Medicine, Jiangsu Academy of Agricultural Sciences, Nanjing, Jiangsu, China; ^2^College of Veterinary Medicine, Nanjing Agricultural University, Nanjing, Jiangsu, China; ^3^GuoTai (Taizhou) Center of Technology Innovation for Veterinary Biologicals, Taizhou, Jiangsu, China

**Keywords:** CRISPR/Cas12a, portable, rabbit hemorrhagic disease virus (RHDV), RT-LAMP

## Abstract

Rabbit hemorrhagic disease is a highly contagious and acute fatal disease caused by rabbit hemorrhagic disease virus (RHDV). The first outbreak of RHDV2 in 2020 has posed a serious threat to the rabbit breeding industry in China. An effective and specific detection strategy for RHDV GI.1 (RHDV1) and GI.2 (RHDV2) is urgently needed. In this study, we established a reverse transcription loop-mediated isothermal amplification (RT-LAMP)-CRISPR/Cas12a-based dual readout portable detection platform. The platform showed excellent specificity to identify RHDV1 and RHDV2 strains and no cross-reaction with other prevalent pathogens of rabbit. The detection limit for RHDV1 and RHDV2 by RT-LAMP-CRISPR/Cas12a could reach 10 copies/μl of the VP60 gene per reaction. Furthermore, 74 clinical samples were detected for both RHDV1 and RHDV2. RT-LAMP-CRISPR/Cas12a-based dual readout portable detection platform showed 25.68% (19/74) RHDV1-positive samples, 43.24% (32/74) RHDV2-positive samples, and 8.11% (6/74) RHDV1/RHDV2 double positive samples, respectively. The coincidence rates of detection RHDV1 and RHDV2 between RT-LAMP-CRISPR/Cas12a and quantitative real-time-polymerase chain reaction (qPCR) were both 97.30%. RT-LAMP-CRISPR/Cas12a showed higher sensitivity and detection rate compared with qPCR. Moreover, the results were visible to the naked eye within 1.5 h combined with lateral flow strips (LFSs) and visual fluorescence. The RT-LAMP-CRISPR/Cas12a portable platform has the advantages of high sensitivity, specificity, fast, low equipment requirements, which can be used in clinical practice in rural areas and resource-limited settings.

## 1. Introduction

Rabbit hemorrhagic disease (RHD) is an acute, violent and highly contagious infection caused by rabbit hemorrhagic disease virus (RHDV), which mainly infects rabbits and hares. Histopathologic features of RHD are significant liver damage, serious coagulation dysfunction, multiple organ edema, congestion, and bleeding [1]. Being globally prevalent, RHD stands as a foremost and severe infectious disease within the rabbit community, inflicting substantial economic losses on the rabbit industry [2–4].

RHDV is a nonenveloped single-stranded positive-sense ribonucleic acid (RNA) virus of the genus Lagovirus, belonging to the Caliciviridae family. Its genomic RNA size is about 7.5 kb with two open reading frames (ORFs). The capsid protein VP60 is encoded by ORF1 [4–6]. Based on the phylogenetic analysis of VP60 sequence, RHDV can be divided into two genomes (GI and GII), which can be subdivided into six genotypes (GI.1, GI.2, GI.3, GI.4, GII.1, and GII.2). According to the latest genome classification, the genomes of “classical RHDV” that were isolated prior to 2010 (including RHDV G1-G6) have been reclassified as GI.1. Additionally, the newly identified RHDV-related virus named RHDV2, which first appeared in France in 2010, has now been categorized as GI.2. Compared with the classical RHDV (RHDV1), RHDV2 demonstrates an expanded host range, infecting both adult and young rabbits across various species [5, 7]. The traditional vaccine exhibits only partial cross-protection against the RHDV2 strain due to the significant genetic and antigenic differences between RHDV1 and RHDV2, while there is no treatment available [8]. Therefore, there is an urgent need to develop a rapid, accurate, and sensitive detection platform capable of discerning these two strains.

Currently, reverse transcription-polymerase chain reaction (RT-PCR) [9], fluorescence quantitative PCR [10, 11], haemagglutination test [12], enzyme linked immunosorbent assay (ELISA) [13, 14], and many other detection methods are available. Immunological detection methods such as the haemagglutination test and ELISA test are convenient, but there are shortcomings such as difficulty working during the window phase and inferior detection accuracy. Meanwhile, pathogen detection methods, such as fluorescence quantitative PCR and RT-PCR, are more accurate, sensitive, and specific, but they still need specialized equipment. These are typically laboratory tests that cannot provide rapid detection strategies in the field. Consequently, these methods might not be able to meet the needs of grass-roots farms for detection.

Loop-mediated isothermal amplification (LAMP) is a widely utilized technology for isothermal amplification at present. With a water bath pot, LAMP enables rapid amplification with superior efficiency compared to traditional PCR methods [15]. It has been applied to the detection of various viruses, such as African swine fever virus (ASFV) [16], Zaire Ebola virus (ZEBOV) [17], Peste des petits ruminants virus (PPRV) [18], human immunodeficiency virus (HIV) [19], and Zika virus (ZIKV) [20]. Moreover, LAMP can be visualized with dyes for on-site quick testing [21]. The potential for false-positive results is LAMP's major flaw, which restricts its use in virus detection [22].

CRISPRs/Cas (clustered regularly interspaced short palindromic repeats and CRISPR-associated proteins) systems exhibit the ability of cis-cleavage, which means they can identify and cleave nucleic acid sequences. Some of them exhibit nonspecific trans-cleavage activity, which means they can identify target sequences with precision while cleaving nontarget sequences [23]. Based on this property, multiple CRISPR/Cas-based nucleic acid detections have been developed, such as SHERLOCK (specific high-sensitivity enzymatic reporter unlocking), DETECTR (DNA endonuclease-targeted CRISPR trans reporter), and HOLMES (a 1-h low-cost multipurpose highly efficient system) [24–26]. Additionally, it is the most widely used technique when combined with isothermal amplification to improve detection specificity. The Cas12a protein binds to guide RNA (gRNA) and subsequently cleavages to the target sequence. This process not only specifically cleaves the target nucleic acid but also indiscriminately cleaves the nearby single-stranded DNA (ssDNA) [27]. If fluorescein and quenching groups are labeled to the ends of ssDNA, after being cleaved, the fluorescent group emits light, which can point to a positive result. Furthermore, the LAMP-CRISPR/Cas12a-based detection platform can be built into a portable one by associating it with lateral flow strips (LFSs) [28].

The portable RHDV1/RHDV2 detection platform based on reverse transcription loop-mediated isothermal amplification (RT-LAMP) and CRISPR/Cas12a technology established in this study aims to detect and distinguish RHDV1 and RHDV2 with high specificity and sensitivity in a short time. A LFS or visible fluorescence can be used to read the detection results. This portable dual readout platform can cater to the demands of grass-roots detection, on-site emergency detection, and other scenarios, which may contribute to the prevention and control of RHD epidemics.

## 2. Materials and Methods

### 2.1. Reagents and Materials

All primers, ssDNA reporters, and gRNAs used in this study were purchased from GenScript (Nanjing, China). WarmStart Multi-Purpose LAMP/RT-LAMP 2X Master Mix (with uracil-DNA glycosylase [UDG]) Protocol, WarmStart Colorimetric LAMP 2X Master Mix Typical LAMP Protocol, EnGen LbaCas12a (Cpf1) were purchased from New England Biolabs (Massachusetts, USA). SYTO 9 Green Fluorescent Nucleic Acid Stain was purchased from Thermo Fisher Scientific (Massachusett, USA). Recombinant RNAse inhibitors were purchased from Takara Bio Inc. (Shiga, Japan). RNA extraction kit was purchased from Vazyme (Nanjing, China). LFSs were purchased from Warbio (Nanjing, China). The seven pathogens involved in the experiments (RHDV1, RHDV2, *Pasteurella multocida*, *Escherichia coli*, *Klebsiella pneumoniae*, *Salmonella typhi*, and Rotavirus) were kept by our laboratory. Fluorescence signals were monitored by QuantStudio 1 (Applied Biosystems, USA). Fluorescence visualization was achieved using a blue light instrument (LABGIC, Beijing, China).

### 2.2. Design of RT-LAMP Primers

The RT-LAMP primers included two outer primers (F3 and B3), two inner primers (forward inner primer [FIP] and backward inner primer [BIP]), and possibly loop primers (loop primer forward [LF] and loop primer backward [LB]) [29]. Three sets of primers targeting the conserved VP60 fragments of RHDV1 (isolate WF2007, GenBank Accession: FJ794180) and RHDV2 (isolate SC2020/04, GenBank Accession: MT383749), respectively, were designed through the LAMP primer online design website (http://primerexplorer.jp/e/) (Supporting Information 1: Table S1). Isolates WF2007 and SC2020/04 were representative strains of RHDV1 and RHDV2 in China, respectively. Subsequently, the primers were screened to select the most efficient one while ensuring no cross-reaction occurred between RHDV1 and RHDV2 detection systems.

### 2.3. Establishment of RT-LAMP Amplification for RHDV1 and RHDV2

Twenty five microliters RT-LAMP reaction system contained 12.5 μl 2 × RT-LAMP master mix, 2 μl RNA, 8 μl enzyme free water and 2.5 μl primer mix. The primer mix is a mixture of inner, outer, and loop primers. The reaction conditions were optimized by fluorescence amplification curves after adding SYTO 9 to the reaction system, including reaction temperature, time, outer and inner primer ratio, loop primer concentration, etc. Additionally, the RT-LAMP analysis can be visualized by introducing neutral red dye into the reaction system.

### 2.4. Establishment of RT-LAMP-CRISPR/Cas12a Detection Platform for RHDV1 and RHDV2

The gRNAs were designed based on the product sequence of RT-LAMP assay (Supporting Information 2: Table S2). The fluorescence quenching ssDNA reporter was 5′-FAM-TTATT-BHQ1-3′. gRNA screening and optimization of Cas12a protein concentration were performed by fluorescence generation from CRISPR/Cas12a cleavage detection. Twenty microliters CRISPR/Cas12a cleavage reaction combined 2 μl RT-LAMP amplification product, 0.5 U/μl RNase inhibitor, 500 nM gRNA, 2 μl NEBuffer 2.1, 250 nM ssDNA reporter, and 1 μl Cas12a protein. Cas12a protein was optimized in the range of 50 to 250 nM as working concentrations. The products of RT-LAMP were used as templates for CRISPR/Cas12a cleavage experiments to verify the trans-cleavage activity of Cas12a. Reactions were incubated at 37°C for 30 min. The fluorescence signal of Cas12a protein trans-cleavage activity and cross-inversion between RHDV1 and RHDV2 was collected per minute and measured with QuantStudio 1.

### 2.5. Combination of the RT-LAMP-CRISPR/Cas12a Detection Platform With LFSs

To establish a portable detection platform, the fluorescence quenching ssDNA (FQ-ssDNA) reporter in the CRISPR/Cas12a cleavage experiment was replaced with a biotin ssDNA reporter (5′ 6-FAM-TTATT-Biotin 3′) (FB-ssDNA). Results were read out using a CRISPR/Cas12a-specific LFS with gold nanoparticle (AuNP)-labeled mouse anti-carboxyfluorescein (FAM) antibody on the sample pad. The control line (C-line) was labeled streptavidin. The test line (T-line) was labeled with anti-mouse immunoglobulin G (IgG) antibody. The FAM-modified end of the ssDNA was conjugated with AuNP on the sample pad. While Cas12a did not cleave the ssDNA reporter, streptavidin in the C line would bind to biotin on the ssDNA and capture it, resulting in a colored C-line indicating a negative result. In contrast, when Cas12a exhibited its activated trans-cutting ability and indiscriminately cleaved the ssDNA reporter, the disconnected FAM-modified end migrated upward. Subsequently, the antimouse IgG antibody on the T-line bound to AuNP-conjugated mouse antibody and captured it, leading to color development at the T-line indicating a positive result.

### 2.6. Methodology Evaluation of RT-LAMP-CRISPR/Cas12a Detection Platform

**Figure 1:**
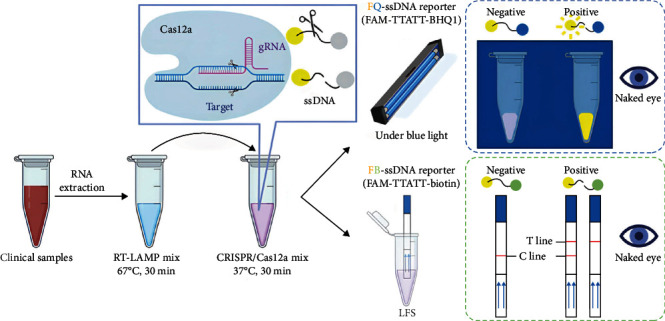
Workflow diagram of RT-LAMP-CRISPR/Cas12a detection platform. The extracted RNA was amplified using RT-LAMP, and the product served as a template for CRISPR/Cas12a cleavage experiments. If a fluorescence quenching probe is used, the results of the CRISPR/Cas12a reaction system can be observed with a blue light instrument. Alternatively, if a biotin probe is employed, the results can be displayed using colloidal gold test strips. CRISPR/Cas, clustered regularly interspaced short palindromic repeat and CRISPR-associated proteins; gRNA, guide RNA; LFS, lateral flow strip; ssDNA, single-stranded DNA.

The workflow diagram of the RT-LAMP-CRISPR/Cas12a detection platform established here is shown in Figure 1. Specificity and sensitivity tests were parts of this detection platform's methodological assessment. Several rabbit-prevalent pathogens were used to measure specificity, including *P. multocida*, *E. coli*, *K. pneumoniae*, *Salmonella typhi* and Rotavirus. To test sensitivity, the full-length VP60 gene of RHDV1 isolate WF2007 and RHDV2 isolate SC2020 were cloned into the pMD18-T vector to construct recombinant plasmids, namely pMD18-T-WF2007-VP60 and pMD18-T-SC2020-VP60, served as targets for assessing the limit of detection (LOD) of RHDV1 and RHDV2.

### 2.7. Comparison of RT-LAMP-CRISPR/Cas12a With Quantitative Real-Time-Polymerase Chain Reaction (qPCR) by Detecting Clinical Samples

Rabbit livers were collected from different regions and farms, then sheared the same sites as clinical samples for RNA extraction. These clinical samples were assessed by both RT-LAMP-CRISPR/Cas12a and qPCR [11]. The results obtained by RT-LAMP-CRISPR/Cas12a were also shown by visual fluorescence and LFS analysis. Afterward, the compliance and sensitivity between these two assays were compared by analyzing the detection rates of RHDV1 and RHDV2.

### 2.8. Statistical Analysis

All data underwent statistical analysis using GraphPad Prism 8.0, employing one-way analysis of variance (ANOVA) to compare different groups. The drawing format of partial figures was referenced in previous research [30]. Each experiment was repeated at least three times, and results were expressed as mean ± standard deviation (SD). Statistical significance was determined at *p* < 0.05.

## 3. Results

### 3.1. Establishment of RT-LAMP Detection Platform for RHDV1 and RHDV2

**Figure 2:**
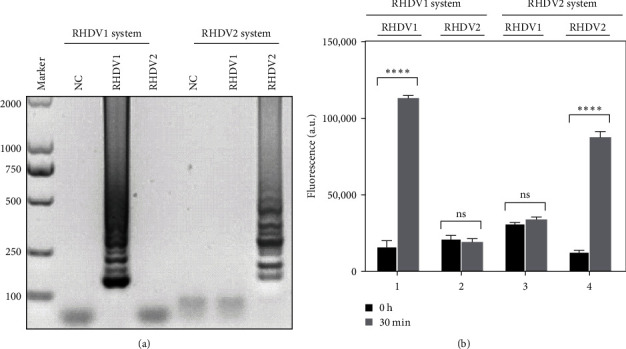
Cross-test of RT-LAMP assay for amplifying RHDV1 and RHDV2. (A) The amplification results visualized by gel electrophoresis. (B) The endpoint fluorescence value of the cross-test carried by fluorescent RT-LAMP. Values are presented as means ± SD (error bars) (*n* = 3 replicates; ⁣^*∗∗∗∗*^*p*  < 0.0001). NC, negative control; ns, no significance; SD, standard deviation.

RT-LAMP amplification reactions were performed using 250 nM SYTO9. Then, primer set 3 for RHDV1 and primer set 2 for RHDV2 were selected as the optimal primers (Supporting Information 3: Figure S1). The primer sequences are shown in Table 1. We used the RHDV1 reaction system to amplify RHDV1 and RHDV2 viruses. Typical ladder strips of LAMP were obtained for RHDV1 and no strip for RHDV2 as expected. Similarly, when we used RHDV2 reaction system to amplify RHDV1 and RHDV2 viruses, ladder strips were obtained for RHDV2 and no strip for RHDV1 (Figure 2A). Cross-test with fluorescent RT-LAMP also showed a good specificity for both RHDV1 and RHDV2 reaction system (Figure 2B). We also used the fluorescent RT-LAMP assay to optimize reaction conditions. The optimal temperature was 67°C for both RHDV1 and RHDV2 (Supporting Information 3: Figure S2). The optimized inner primer to outer primer ratio is 1:6 (200 nM:1200 nM) for RHDV1 (Supporting Information 3: Figure S3) and 1:4 (200 nM:800 nM) for RHDV2 (Supporting Information 3: Figure S3). The optimal loop primer concentrations were 600 nM for both RHDV1 and RHDV2 (Supporting Information 3: Figure S4). Neutral red dye was added to enable results visualization with a distinct color between positive as yellow and negative as pink (Supporting Information 3: Figure S1).

### 3.2. Establishment of RT-LAMP-CRISPR/Cas12a Detection Platform for RHDV1 and RHDV2

**Figure 3:**
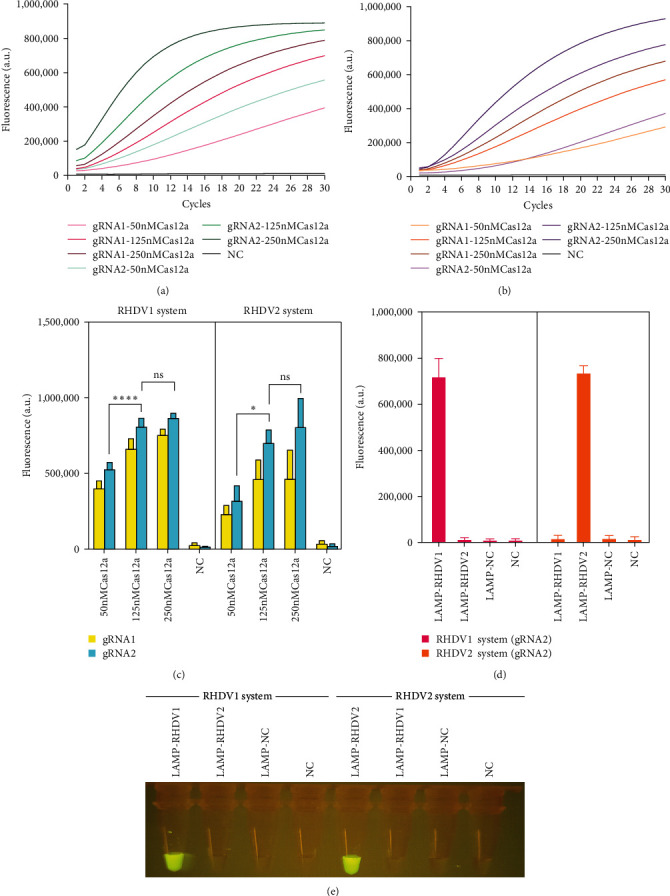
Establish RT-LAMP-CRISPR/Cas12a detection method for RHDV1 and RHDV2. (A) The fluorescence value curve of orthogonal test for gRNA screening and cas12a protein concentration optimization in RHDV1 detection system. (B) The fluorescence value curve of the orthogonal test for gRNA screening and cas12a protein concentration optimization in RHDV2 detection system. (C) Comparison of fluorescence values at the endpoint of orthogonal test (*n* = 3 replicates, values are presented as means ± SD; ⁣^*∗∗∗∗*^*p*  < 0.0001, ⁣^*∗*^*p*  < 0.05). (D) Verify the trans-cleavage activity of cas12a protein and cross-reaction of CRISPR/Cas12a detection for RHDV1 and RHDV2. (E) The results of trans-cleavage activity verification and cross-test under blue light. CRISPR/Cas, clustered regularly interspaced short palindromic repeat and CRISPR-associated proteins; gRNA, guide RNA; NC, negative control; ns, no significance; RHDV, rabbit hemorrhagic disease virus; SD, standard deviation.

We designed two gRNAs for RHDV1 and RHDV2, respectively. gRNA2 showed a higher fluorescence signal with the efficiency of cleavage activity for RHDV1 and RHDV2 (Figure 3A,B) (Table 2). To detect the optimal concentration of Cas12a protein, we used 50, 125 and 250 nM to test the fluorescence level. Results indicated that 125 nM Cas12a protein could produce significantly higher level of fluorescence signal compared with 50 nM group (*p*  < 0.05). However, there was no significant difference compared to 250 nM group (Figure 3C). Therefore, optimal concentration of Cas12a protein was set as 125 nM.

The selected gRNAs were used for Cas12a protein trans-cleavage activity validation and cross-testing between the RHDV1 and RHDV2 reaction systems. When we detected RHDV1 strain using RHDV1 reaction systems, the results demonstrated a significant increase in fluorescence value (*p*  < 0.0001) compared to other groups (Figure 3D). Same results were obtained for RHDV2 reaction systems (Figure 3D). Visualizations showed the same results observed under blue light (Figure 3E). It suggested that the fluorescence generated was derived from the trans-cleavage activity of Cas12a protein and no cross-reactivity between RHDV1 and RHDV2 systems.

### 3.3. Specificity Assessment of RT-LAMP-CRISPR/Cas12a Detection Platform for RHDV1 and RHDV2

**Figure 4:**
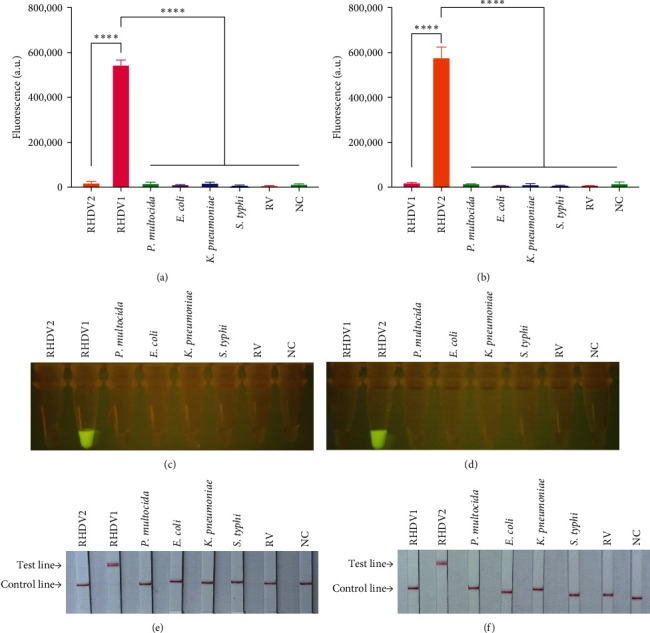
Specificity evaluation of RT-LAMP-CRISPR/Cas12a detection platform. (A, B) Evaluate the specificity of RT-LAMP-CRISPR/Cas12a fluorescent detection platform by fluorescence values at the endpoint (*n* = 3 replicates, values are presented as means ± SD; ⁣^*∗∗∗∗*^*p*  < 0.0001). (C, D) Specificity evaluation of RT-LAMP-CRISPR/Cas12a-based visual fluorescence detection platform under blue light. (E, F) Specificity evaluation of RT-LAMP-CRISPR/Cas12a-based LFS detection platform (A, C, E stands for RHDV1 detection system; B, D, F stands for RHDV2 detection system). CRISPR/Cas, clustered regularly interspaced short palindromic repeat and CRISPR-associated proteins; LFS, lateral flow strip; NC, negative control; RHDV, rabbit hemorrhagic disease virus; SD, standard deviation.

We used RT-LAMP-CRISPR/Cas12a detection platform to detect rabbit pathogens including RHDV1, RHDV2, *P. multocida*, *E. coli*, *K. pneumoniae*, *Salmonella typhi* and Rotavirus. The tested rabbit pathogens showed negative results except for RHDV1 detecting using RHDV1 system or RHDV2 detecting using RHDV2 system (Figure 4A,B). Incidentally, except RHDV1 or RHDV2, the detection for other main rabbit pathogens by visualization analysis and LFSs was also negative (Figure 4C–F), indicating the RT-LAMP-CRISPR/Cas12a detection platform for RHDV1 and RHDV2 possessed good specificity.

### 3.4. Sensitivity Assessment of RT-LAMP-CRISPR/Cas12a Detection Platform for RHDV1 and RHDV2

**Figure 5:**
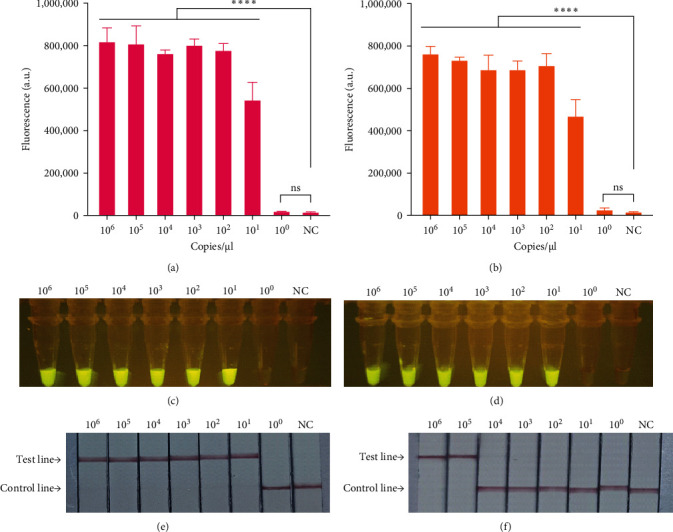
Sensitivity evaluation of RT-LAMP-CRISPR/Cas12a detection platform. (A, B) Evaluate the sensitivity of the RT-LAMP-CRISPR/Cas12a fluorescent detection platform by fluorescence values at the endpoint (*n* = 3 replicates, values are presented as means ± SD; ⁣^*∗∗∗∗*^*p*  < 0.0001). (C, D) Sensitivity evaluation of RT-LAMP-CRISPR/Cas12a-based visual fluorescence detection platform under blue light. (E, F) Sensitivity evaluation of RT-LAMP-CRISPR/Cas12a-based LFS detection platform (A, C, E stands for RHDV1 detection system; B, D, F stands for RHDV2 detection system). CRISPR/Cas, clustered regularly interspaced short palindromic repeat and CRISPR-associated proteins; LFS, lateral flow strip; NC, negative control; RHDV, rabbit hemorrhagic disease virus; SD, standard deviation.

VP60 full-length plasmids pMD18-T-WF2007-VP60 and pMD18-T-SC2020-VP60 were used as detection templates for sensitivity tests at a 10-fold ratio dilution (1 × 10^6^ copies/μl-1 × 10°copies/μ l). RT-LAMP-CRISPR/Cas12a assay targeting RHDV1 and RHDV2 could both detect 1 × 10^1^ copies/μ l. Meanwhile, the fluorescence values obtained for detecting 1 × 10° copies/μ l of dsDNA template did not differ substantially from those of the negative control (NC) sample (*p*  > 0.05) (Figure 5A,B). Likewise, comparable results were observed with the naked eye during blue light irradiation (Figure 5C,D) and strip testing (Figure 5E,F).

### 3.5. Detection of RHDV1 and RHDV2 in Clinical Samples With RT-LAMP-CRISPR/Cas12a Portable Platform and qPCR

**Figure 6:**
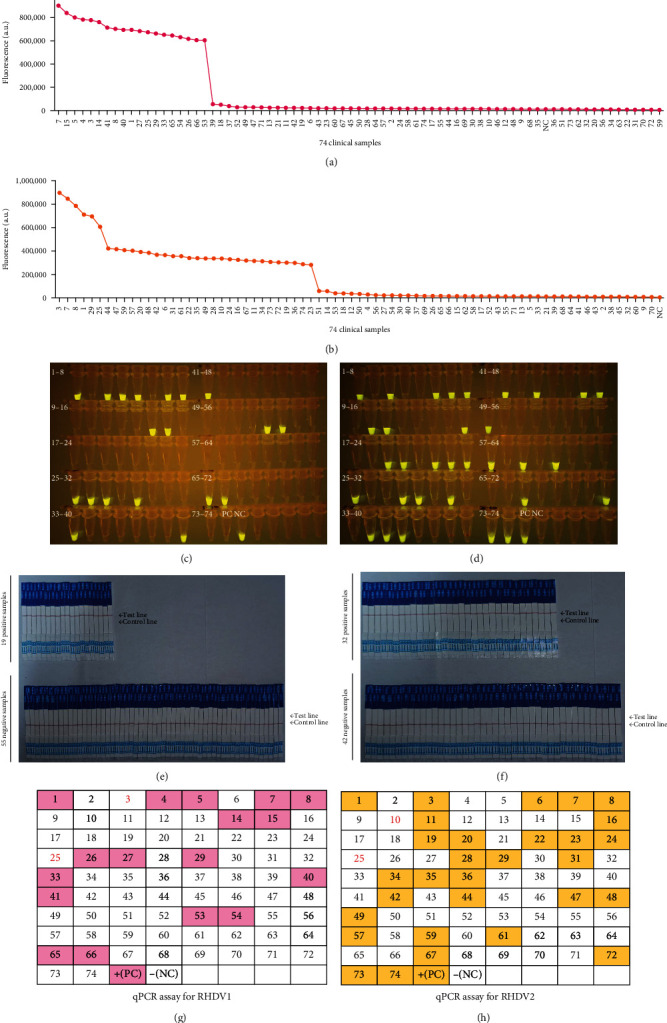
Clinical utility performance results of RT-LAMP-CRISPR/Cas12a detection platform. (A, B) The endpoint fluorescence value of 74 clinical samples detected by RT-LAMP-CRISPR/Cas12a fluorescence detection platform (the critical values were 55,482 and 57,905, respectively). (C, D) The results of 74 clinical samples detected by RT-LAMP-CRISPR/Cas12a-based visualization platform were interpreted by naked eye observation under blue light. (E, F) The results of 74 clinical samples detected by RT-LAMP-CRISPR/Cas12a-based visualization platform was interpreted by naked eye observation using lateral flow strips. (G, H) The results of 74 clinical samples detected by qPCR method, pink/yellow means positive, white means negative, and the red number indicates that the detection result of this sample is inconsistent with the method established in this study. CRISPR/Cas, clustered regularly interspaced short palindromic repeat and CRISPR-associated proteins; NC, negative control.

74 clinical samples were tested using both RT-LAMP-CRISPR/Cas12a portable platform and qPCR for RHDV1 and RHDV2. Results showed that 19 samples were RHDV1-positive using RT-LAMP-CRISPR/Cas12a portable platform with a detection rate of 25.68% (Figure 6A,C,E), while 17 samples were RHDV1-positive using qPCR with a detection rate of 22.97% (Figure 6G). Thirty-two samples were RHDV2-positive using RT-LAMP-CRISPR/Cas12a portable platform with a detection rate of 43.24% (Figure 6B,D,F), while 30 samples were RHDV2-positive using qPCR with a detection rate of 40.54% (Figure 6H). The coincidence rates of RT-LAMP-CRISPR/Cas12a portable platform and qPCR were 97.30% for both RHDV1 and RHDV2. Among these 74 samples, six (numbered 1, 3, 7, 8, 25, and 29) and four (numbered 1, 7, 8, and 29) mixed infection samples, which were infected with both RHDV1 and RHDV2, were detected using RT-LAMP-CRISPR/Cas12a portable platform and qPCR, resulting in detection rates of 8.11% and 5.41%, respectively. The direct visual observation of fluorescent and LFS by the naked eye yielded consistent results for both detection systems, thereby mutually validating the two readout strategies. The results showed RT-LAMP-CRISPR/Cas12a portable platform was more sensitive than qPCR. Furthermore, RT-LAMP-CRISPR/Cas12a platform can be regarded as a new diagnostic method for detecting and distinguishing RHDV1 and RHDV2, which is feasible in clinical practice.

## 4. Discussion

RHDV causes high mortality rates (up to 90%) in rabbits, which is a serious threat to rabbitry worldwide. RHDV2 discovered in France in 2010 is genetically and antigenically different from classical RHDV (RHDV1) [31]. The first outbreak of RHDV2 in China resulted in huge financial losses in 2020. Therefore, effective and accurate diagnostic tools that can distinguish between these two strains are especially crucial for the prevention and control of RHD outbreaks. PCR, qPCR and other techniques are effective tools for detecting RHDV. However, the requirements of lab equipment and labors make them challenge to be applied for rapid detection at the grassroots level. In this study, we established a simple and highly sensitive RT-LAMP-CRISPR/Cas12a rapid detection platform for RHDV1 and RHDV2. The results can be read out directly by both visual fluorescence and LFSs.

LAMP analysis has been used for the detection of a variety of pathogens. Four to six primers are needed for LAMP to direct the amplification, and one of the most crucial aspects of optimizing the LAMP reaction is primer design and selection [32]. Here, three sets of primers were designed according to the conserved fragment VP60 of RHDV1 and RHDV2, respectively, and finally selected the optimal primer sets. In addition, the sensitivity analysis demonstrated that the detection limits of RT-LAMP-CRISPR/Cas12a portable platform were 10 copies/μ l for both RHDV1 and RHDV2. Despite the high sensitivity of LAMP analysis, it is prone to cross-contamination leading to false-positive results. Combining with CRISPR/Cas12a, detection system is effective to avoid false-positive outcomes [33].

Currently, the CRISPR/Cas12a-based detection system is hard to identify low concentrations of pathogenic signals [29], but this limitation can be solved by combining it with LAMP. We designed two gRNAs based on the target fragments amplified by RT-LAMP for RHDV1 and RHDV2, respectively, to select the optimal gRNA. The use of fluorescent quenching reporters allowed the results to be observed directly with the naked eye under blue light irradiation. We also used biotin reporters together with LFSs for readout. The detection limits for the visualization and LFSs were both 10 copies/μ l. No other rabbit pathogens were detected by RHDV1 or RHDV2 detection systems indicating RT-LAMP-CRISPR/Cas12a platform possessed good specificity. The results of 74 clinical samples showed the developed platform had a high compliance rate with qPCR. It also was more sensitive than qPCR. To reduce the effect of aerosol pollution, we added UDG enzyme into RT-LAMP reaction system. We also tested the major Chinese RHDV epidemic strains, and all of them could be detected by the developed platform. For on-site detection, we tested kinds of nucleic acid releasing agents and the HUDSON method for rapid extraction of nucleic acid [34]. The virus could still be detected but with a lower detection rate. Developing an appropriate nucleic acid extraction method will be done in the future. Finally, RT-LAMP-CRISPR/Cas12a platform developed in this study can be used for the rapid, low-cost, sensitive, and portable detection of RHDV1 and RHDV2. Besides, Sharma, Kabir, and Asghar [35] developed a fully automated microfluidic experimental platform in conjunction with LAMP, which ensured the target nucleotide fragments adsorbed on the surface of magnetic beads could be purified and amplified by precisely controlling the movement of magnetic beads. There are new technologies that combine LAMP with lateral flow biosensors (LFBs) [36] and electrochemical technology [37]. The CRISPR/Cas system can also be used in conjunction with recombinase polymerase amplification (RPA), rolling circle amplification (RCA), nucleic acid sequence-based amplification (NASBA), and other technologies [38–40]. In the future, we can also seek better solutions by combining these assays.

## Figures and Tables

**Table 1:** Primers for RT-LAMP.

Virus	Primer	Sequence
RHDV1	F3	AACGGCAGCACATATGGC
	B3	GCTGTTAAAGGGCACGAATG
	FIP	ACGTTGGTGGAGTTGTTCCCAGTTTGCCGACATTGACCATCG
	BIP	GGTACGCTAATGCTGGGTCTGCATGTCAGGGAAGCCGTCT
	LB	GATTGACAACCCTATCTCCCAGGTT

RHDV2	F3	ATGCTAGTGCCGGGTCTG
	B3	GTTGCTCGGTACTCCAGTG
	FIP	GGTAGGGATGGTGATACCGCTGAACCCCATCTCCCAAATTGC
	BIP	GGTCGGGTTCGGTGGGATCTTAAGCCTGCATGGTCGTGA
	LF	TGACATGTCAGGGAAACCATCTGG
	LB	AACAGCAGTAATGGTGCCCCC

Abbreviations: BIP, backward inner primer; FIP, forward inner primer; LB, loop primer backward; LF, loop primer forward; RHDV, rabbit hemorrhagic disease virus; RT-LAMP, reverse transcription loop-mediated isothermal amplification.

**Table 2:** gRNA sequences.

Virus	PAM	Sequence
RHDV1	TTTG	UAAUUUCUACUAAGUGUAGAUGUACGCUAAUGCUGGGUCUG
RHDV2	TTTC	UAAUUUCUACUAAGUGUAGAUCCUGACAUGUCAUUUGUACCC

Abbreviations: gRNA, guide RNA; PAM, protospacer adjacent motif; RHDV, rabbit hemorrhagic disease virus.

## Data Availability

All other materials are available from corresponding authors (Fang Wang and Yanhua Song).

## Supporting Information

Supporting Information 1 Table S1: Primers for RT-LAMP.

Supporting Information 2 Table S2: guide RNA (gRNA) sequences.

Supporting Information 3 Figure S1: Application plot of fluorescent RT-LAMP assay with three primer sets designed for RHDV1 (A) and RHDV2 (B). Figure S2: Optimize the reaction temperature for RHDV1 (A) and RHDV2 (B) with fluorescent RT-LAMP assay. The RT-LAMP assay was performed under 61, 63, 65, 67, and 69°C reaction temperature, respectively. Figure S3: Optimize the outer and inner primer concentration ratios for RHDV1 (A) and RHDV2 (B) with fluorescent RT-LAMP assay. The concentration ratio of outer primer and inner primer with 200:400, 200:800, 200:1200, 200:1600, and 200:2000 nM was used to perform fluorescent RT-LAMP assay, respectively. Figure S4: Optimize the loop primer concentration for RHDV1 (A) and RHDV2 (B) with fluorescent RT-LAMP assay. The concentration of loop primer with 200, 400, 600, 800, and 1000 nM was used to perform fluorescent RT-LAMP assay, respectively. Figure S5: Visual RT-LAMP results with neutral red (pink represents negative, yellow represents positive). The above supporting information are shown in the attachment Supplementary materials.pdf.
