# Supplementary material for: Specific Detection of RHDV GI.1 and GI.2 by RT-LAMP-CRISPR/Cas12a Platform
Source: Transbound Emerg Dis. 2024 Nov 19;2024:3881457. doi: 10.1155/tbed/3881457 (PMC12020271; doi:10.1155/tbed/3881457)
Supplement: Supporting Information 1 — Table S1: Primers for RT-LAMP. [file 3881457.f1.docx]

Supplementary table1. Primers for RT-LAMP.

| Virus | Set | Primer | Sequence (5’ → 3’) |
| --- | --- | --- | --- |
| RHDV1 | ① | F3 | TCCCTGACATGTCATTCGTG |
|  |  | B3 | GGTTTGTGCCGGTTACCAC |
|  |  | FIP | CGGGGGCACCGTTGTTACTGTTAACAGCCCCAACATTCCG |
|  |  | BIP | AGTTAGGTTTTGCCACTGGGGCCAGTCTGTGCACCTGAAGTG |
|  |  | LF | ACCACCAAACCCGACCCAC |
|  |  | LB | AACAGCCTCCAGCCCACCA |
|  | ② | F3 | GCATGCAGTTCCGCTTCA |
|  |  | B3 | ACTAGTGTGGGGACAAGGC |
|  |  | FIP | CTCCAACCCTGGCCCAATCTCGTGTGTTTGGTGGGCGAC |
|  |  | BIP | CGCCCGTTCACTCGAACCTGCAGGGTCACCAGTTGGATG |
|  |  | LF | CCTGGTGGTATCACAGCCGC |
|  |  | LB | TCACCATGCCAGACTTGCGT |
| RHDV2 | ① | F3 | ACCACCGGGCATTGAGAT |
|  |  | B3 | TCGTGGATCCACCAAATGG |
|  |  | FIP | TGACTGGTTCGAGTGAACGAGCTGGGCCAGGTTTGGAAGT |
|  |  | BIP | GCGCCCCAACATGTACCACCAACGCTCAGGACCAACGT |
|  |  | LF | TGACAACATGAGGGAATTGTCTG |
|  |  | LB | CAACAGGCAACCCTGGC |
|  | ③ | F3 | CATGTACCACCCAACAGGC |
|  |  | B3 | GTGAGAACTGGGGTTGTGAG |
|  |  | FIP | CTCGTGGATCCACCAAATGGGT-GTTCCCACGTTGGTCCTG |
|  |  | BIP | AGTTTGTGATGATCCGTGCCCC-GAGATCTGCGGGCGAGAT |
|  |  | LF | TGATGAGGTTGTTGTAAACGCT |
|  |  | LB | TCCAGTAAGACCGTTGACTCG |
