# Supplementary material for: Specific Detection of RHDV GI.1 and GI.2 by RT-LAMP-CRISPR/Cas12a Platform
Source: Transbound Emerg Dis. 2024 Nov 19;2024:3881457. doi: 10.1155/tbed/3881457 (PMC12020271; doi:10.1155/tbed/3881457)
Supplement: Supporting Information 2 — Table S2: guide RNA (gRNA) sequences. [file 3881457.f2.docx]

Supplementary table 2. gRNA sequences.

| Virus | gRNA | PAM | Sequence |
| --- | --- | --- | --- |
| RHDV1 | gRNA1 | TTTG | UAAUUUCUACUAAGUGUAGAUCCGACAUUGACCAUCGAAGA |
| RHDV2 | gRNA1 | TTTG | UAAUUUCUACUAAGUGUAGAUUACCCUUCAGCGGUAUCACCA |
