# Supplementary material for: Specific Detection of RHDV GI.1 and GI.2 by RT-LAMP-CRISPR/Cas12a Platform
Source: Transbound Emerg Dis. 2024 Nov 19;2024:3881457. doi: 10.1155/tbed/3881457 (PMC12020271; doi:10.1155/tbed/3881457)
Supplement: Supporting Information 3 — Figure S1: Application plot of fluorescent RT-LAMP assay with three primer sets designed for RHDV1 (A) and RHDV2 (B). Figure S2: Optimize the reaction temperature for RHDV1 (A) and RHDV2 (B) with fluorescent RT-LAMP assay. The RT-LAMP assay was performed under 61, 63, 65, 67, and 69°C reaction temperature, respectively. Figure S3: Optimize the outer and inner primer concentration ratios for RHDV1 (A) and RHDV2 (B) with fluorescent RT-LAMP assay. The concentration ratio of outer primer and inner primer with 200:400, 200:800, 200:1200, 200:1600, and 200:2000 nM was used to perform fluorescent RT-LAMP assay, respectively. Figure S4: Optimize the loop primer concentration for RHDV1 (A) and RHDV2 (B) with fluorescent RT-LAMP assay. The concentration of loop primer with 200, 400, 600, 800, and 1000 nM was used to perform fluorescent RT-LAMP assay, respectively. Figure S5: Visual RT-LAMP results with neutral red (pink represents negative, yellow represents positive). The above supporting information are shown in the attachment Supplementary materials.pdf. [file 3881457.f3.docx]

**Supplementary Figures**

**FIGURE S1** Application plot of fluorescent RT-LAMP assay with 3 primer sets designed for RHDV1(A) and RHDV2(B).

**FIGURE S2** Optimize the reaction temperature for RHDV1(A) and RHDV2(B) with fluorescent RT-LAMP assay. The RT-LAMP assay was performed under 61°C, 63°C, 65°C, 67°C and 69°C reaction temperature, respectively.

**FIGURE S3** Optimize the outer and inner primer concentration ratios for RHDV1(A) and RHDV2(B) with fluorescent RT-LAMP assay. The concentration ratio of outer primer and inner primer with 200 nM:400 nM, 200 nM:800 nM, 200 nM:1200 nM, 200 nM:1600 nM, 200 nM:2000 nM was used to perform fluorescent RT-LAMP assay, respectively.

**FIGURE S4** Optimize the loop primer concentration for RHDV1(A) and RHDV2(B) with fluorescent RT-LAMP assay. The concentration of loop primer with 200 nM, 400 nM, 600 nM, 800 nM, and 1000 nM was used to perform fluorescent RT-LAMP assay, respectively.

**FIGURE S5** Visual RT-LAMP results with neutral red (pink represents negative, yellow represents positive).

**Supplementary Tables**

**Supplementary Table 1. Primers for RT-LAMP**

**Supplementary Table 2. gRNA sequences**
